# Synthesis and Characterization of MnO_2_@Cellulose and Polypyrrole-Decorated MnO_2_@Cellulose for the Detection of Chemical Warfare Agent Simulant

**DOI:** 10.3390/ma15207313

**Published:** 2022-10-19

**Authors:** Sanjeeb Lama, Sumita Subedi, Sivalingam Ramesh, Kyeongho Shin, Young-Jun Lee, Joo-Hyung Kim

**Affiliations:** 1 Laboratory of Intelligent Devices and Thermal Control, Department of Mechanical Engineering, Inha University, Incheon 22212, Korea; 2 Department of Chemistry and Chemical Engineering, Inha University, Incheon 22212, Korea; 3 Department of Mechanical, Robotics and Energy Engineering, Dongguk University, Seoul 04620, Korea

**Keywords:** chemical warfare agents (CWAs), quartz crystal microbalance (QCM), surface acoustic wave (SAW), dimethyl methyl phosphonate (DMMP), volatile compounds (VOCs)

## Abstract

Chemical warfare agents (CWAs) have been threatening human civilization and its existence because of their rapid response, toxic, and irreversible nature. The hybrid nanostructured composites were synthesized by the hydrothermal process to detect the dimethyl methyl phosphonate (DMMP), a simulant of G-series nerve agents, especially sarin. Cellulose (CE), manganese oxide cellulose (MnO_2_@CE), and MnO_2_@CE/polypyrrole (PPy) exhibited a frequency shift of 0.4, 4.8, and 8.9 Hz, respectively, for a DMMP concentration of 25 ppm in the quartz crystal microbalance (QCM). In surface acoustic wave (SAW) sensor, they exhibited 187 Hz, 276 Hz, and 78 Hz, respectively. A comparison between CE, MnO_2_@CE, and MnO_2_@CE/PPy demonstrated that MnO_2_@CE/PPy possesses excellent linearity with a coefficient of determination (COD or R^2^) of 0.992 and 0.9547 in the QCM and SAW sensor. The hybrid composite materials showed a reversible adsorption and desorption phenomenon in the reproducibility test. The response and recovery times indicated that MnO_2_@CE/PPy showed the shortest response (~23 s) and recovery times (~42 s) in the case of the QCM sensor. Hence, the pristine CE and its nanostructured composites were compared to analyze the sensing performance based on sensitivity, selectivity, linearity, reproducibility, and response and recovery times to detect the simulant of nerve agents.

## 1. Introduction

The preferred characteristics of the modern chemical sensor lie in its rapid response, accuracy, precision, stability, and field operations (vacuum and liquid medium) [1]. Many studies have focused on developing portable and array sensors to meet the demand for on-site monitoring [2,3]. To provide alternatives to traditional equipment, the accuracy of these portable devices should be comparable to conventional analytical techniques. There are different types of transducers, namely, piezoelectric, acoustic, resistive, inductive, capacitive, electromagnetic, and electrodynamic [4], that are extensively researched for chemical sensor development. The acoustic wave sensor uses mechanical or acoustic waves as a sensing mechanism. As the acoustic wave passes through or on the surface of the sensing materials, any small changes in the transmission path can directly affect the amplitude and velocity of the propagated acoustic wave [5]. Herein, acoustic wave sensors (surface acoustic waves-based (SAW) and quartz crystal microbalance (QCM) sensors) were used to measure the changes in frequency as the wave propagates.

Chemical warfare agents (CWAs) are potent chemicals that can cause immense damage and annihilate human lives. The use of CWAs on Iraqi civilians (1988) [6], the Tokyo subway (1995) [7], and the Syria war (2013) [8] showed its devastating nature and the need to be detected as early as possible. Nerve agents, such as sarin (military designation: GB), soman (GD), and tabun (GA), see Figure S1, one of the most toxic CWAs, act as an inhibitor of the neurotransmitter enzyme called acetylcholine, disrupting the central nervous system and eventually leading to death [9]. The median lethal concentrations (LCt_50_) of GB and GA are 100 and 400 mg·min/m^3^, respectively [10]. Exposure at these concentrations or beyond can prove fatal to human lives. Because nerve agents are prohibited, DMMP is used as a simulant that imitates the structure of G-series nerve agents and is relatively less toxic.

Several novel nanocomposites, such as metal oxides, polymers, and their hybrid composites, have been introduced to detect the DMMP. Manganese oxide (*M.W.* = 86.937 g/mol) with a density of 5.0 g/cm^3^ has a decomposition temperature of 535 °C [11]. Because of their surface and electronic properties and the gas adsorbent capacity, manganese oxides are used widely in various fields, such as supercapacitors [12], batteries [13], water treatment [14], catalysts [15], decomposition [16], adsorption [17], and gas detection [18,19]. Thomas et al. demonstrated the use of TiO_2_@MnO_2_ nanorod arrays in the microcantilevers to enhance the detection of CWAs and their simulant [20]. Segal et al. reported using amorphous manganese oxide as a catalyst to decompose the simulant of CWAs, DMMP [21]. PPy has been used extensively in gas sensors, batteries [22], supercapacitors [23], corrosion protection [24], and wastewater treatment [25]. Tiwari et al. displayed the use of a conducting polymer—PPy with copper phthalocyanine—to sense the DMMP vapor [26]. Jun et al. exhibited the use of PPy with tin oxide, which was highly sensitive to detect DMMP [27].

In recent years, cellulose has attracted considerable attention because of the notion of environmental protection, and is non-toxic, low-cost, abundant, derived from agricultural products, and biodegradable. Cellulose has been applied to a vast majority of fields, from the textiles to medicine and pharmaceuticals [28]. Pang et al. reported the use of hybrid composites comprising of cellulose, TiO_2_, and conducting polymer PANI to detect ammonia gas from 10–250 ppm at room temperature [29]. Rapid detection of VOCs was observed using ethyl cellulose composite with graphene in the flexible gas sensor with its application in wearable electronics [30].

**Figure 1:**
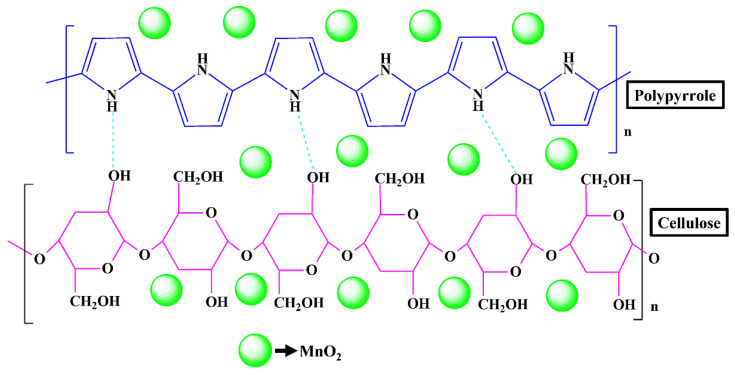
Chemical structure of MnO_2_@CE/PPy. The figure is not to scale.

Despite its numerous advantages, its properties, such as chemical inertness [31], high stiffness and strength [32], and poor solubility and processability [33], make it difficult to use cellulose in the field of sensors. In addition, the complex sensing mechanism involving the cellulose nanocomposite is still unclear and needs to be elucidated [32,34]. To the best of the authors’ knowledge, there are still no reports about incorporating MnO_2_ with the cellulose and conducting polymer PPy to detect CWA simulant at room temperature. Hence, the synthesized MnO_2_@CE and MnO_2_@CE/PPy were compared with pure cellulose to detect the CWA simulant. MnO_2_@CE has proven its wide applicability in wastewater treatment [35], supercapacitors [36], lithium-ion batteries [37], antibacterial activity [38], and formaldehyde degradation [39]. Figure 1 shows the chemical structure of MnO_2_@CE/PPy.

This paper reports the synthesis of hybrid nanostructured composites—MnO_2_@CE, and MnO_2_@CE/PPy—using the hydrothermal process in which cellulose (CE) acts as a base material. The pristine CE and the synthesized composites were characterized by Fourier-transform infrared spectroscopy (FTIR), X-ray powder diffraction (XRD), X-ray photoelectron spectroscopy (XPS), scanning electron microscopy (SEM), and transmission electron microscopy (TEM). CE and its hybrid nanostructured composites were applied to the QCM and SAW sensor, which were compared in terms of the frequency shift, linearity, reproducibility, selectivity, and response and recovery times for the DMMP vapor varying from 25 to 150 ppm. The frequency shifts (Δ*f*) of MnO_2_@CE and MnO_2_@CE/PPy were ~12 times and ~22 times higher than that of pristine cellulose at 25 ppm DMMP, respectively, in the QCM sensor. At 150 ppm, the frequency shifts were ~five times and ~six times higher than the pristine cellulose. The hybrid composites of the MnO_2_@CE/PPy showed an excellent correlation coefficient (R^2^) of 0.992. In addition, MnO_2_@CE/PPy showed the shortest response and recovery times of ~23 s and ~42 s, respectively, at 75 ppm DMMP in the QCM sensor. Finally, the possible adsorption mechanism of DMMP onto the surface of synthesized MnO_2_@CE/PPy is proposed.

## 2. Materials and Methods

### 2.1. Materials

Cellulose (CE) as a microcrystalline powder (20 µm), pyrrole, hydrogen peroxide (H_2_O_2_), iron (III) chloride (FeCl_3_), dimethylformamide (DMF), potassium permanganate (KMnO_4_), manganese (II) acetate (Mn(CH_3_COO)_2_), ammonium hydroxide (NH_4_OH), ethanol, sulfuric acid (H_2_SO_4_), isopropyl alcohol (IPA), methanol, n-hexane, and toluene were purchased from Sigma Aldrich, Seoul, Korea.

### 2.2. Synthesis of MnO_2_@CE and MnO_2_@CE/PPy

#### 2.2.1. Oxidative Polymerization of Pyrrole to Polypyrrole

Pyrrole (6 g), H_2_O_2_ (10 mL), and FeCl_3_ (1.5 g) were mixed with distilled water (300 mL) in a beaker (500 mL) and stirred continuously for four hours at room temperature. The mixture was heated in an oven at 90 °C for two hours to promote oxidative polymerization. Hence, the monomer pyrrole was converted to polymerized polypyrrole (PPy). Thus, the obtained PPy was collected and used further to synthesize MnO_2_@CE/PPy.

#### 2.2.2. Synthesis of MnO_2_@CE

The achieved CE (1 g) was mixed with DMF (10 mL) to produce a CE solution. KMnO_4_ (1.5 g, 0.01 M) and Mn(CH_3_COO)_2_ (1.5 g, 0.01 M) were liquified with distilled water and mixed with the CE solution. The mixture was transferred to a 500 mL beaker. Subsequently, ammonium hydroxide (25 mL) was poured into the mixture. During these mixing processes, the solution was continuously stirred and further stirred for 12 h at 95 °C. The solution was transferred to an oven at 180 °C for 12 h. The solution was purified with distilled water and dried at 95 °C. The resulting product was further calcined at 400 °C for 10 h in a vacuum furnace that was purified by water and ethanol solution consecutively. Finally, the MnO_2_@CE composite was obtained and kept in the cabinet for further use.

#### 2.2.3. Synthesis of MnO_2_@CE/PPy

**Figure 2:**
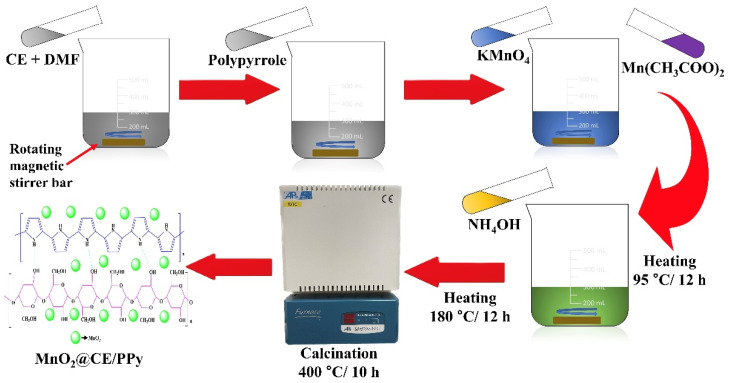
Fabrication process of MnO_2_@CE/PPy.

Similarly, a CE solution was prepared. Urea (1.2 g) was added to 100 mL of distilled water, which was further added to the CE solution. Subsequently, 1.0 g of polymerized polypyrrole, 1.5 g of KMnO_4_ (0.01 M), and 1.5 g of Mn (CH_3_COO)_2_ (0.01 M) were liquefied with distilled water and mixed with a CE solution. A similar process to the above-mentioned was conducted to obtain MnO_2_@CE/PPy. Hence, the collected composite was kept in the cabinet for additional use. Figure 2 shows the fabrication of MnO_2_@CE/PPy.

### 2.3. Fabrication of the Sensor and Coating Process

#### 2.3.1. For the QCM Sensor

A QCM sensor with an AT-cut 5 MHz quartz crystal with Cr/Au electrodes was purchased from the Stanford Research System (SRS). The bare QCM sensor was soaked with a piranha solution (30% H_2_O_2_: 99% H_2_SO_4_; 1:3 *v*/*v*) for 30 min. The treated QCM sensor was washed with pure water and ethanol sequentially. The QCM sensor was kept in an oven at 60 °C for one hour.

Subsequently, CE, MnO_2_@CE, and PPy-grafted MnO_2_@CE were stirred with IPA in a 10 mg:1 mL ratio. The obtained solutions were ultrasonicated for three hours. Hence, 15 µL of the acquired suspension was drop-coated carefully at the center of the treated QCM sensor. The drop-coated suspension was allowed to volatilize naturally. The sensing materials deposited on QCM sensors were heated for one hour at 60 °C in an oven. Finally, the QCM sensors were allowed to cool at a room temperature of 22 ± 2 °C and were used as received.

#### 2.3.2. For the SAW Sensor

The fabrication of the 250 MHz SAW sensor is reported elsewhere [10,19,40,41]. CE, MnO_2_@CE, and MnO_2_@CE/PPy were mixed with IPA at a 1 mg:7 mL ratio because SAW sensors are highly susceptible to the weight of the deposited sensing materials. The ultrasonication process of the mixture was conducted for three hours. Considering the weight of the suspension, 2.5 µL of the solution was drop-coated at the center of the SAW sensor. The coated SAW sensors were allowed to volatilize naturally and kept in an oven at 60 °C for one hour. The sensors were kept at a room temperature of 22 ± 2 °C for cooling and used in the experiment without further processing.

### 2.4. Target Vapor Preparation and Sensor Measurement System

A detailed explanation of targeted vapor generation and sensor measurement systems (vapor generating bubbler, analyte delivery system, detection chamber, and data acquisition) are reported elsewhere [42]. Briefly, the bubbler flask was purchased from DURAN, which has a volume of 500 mL. Figure S2 shows the bubbler flask used in the experiment and a schematic diagram of the vapor-generating process in the bubbler. The resulting bubbler flasks were cleaned several times with ethanol and water consecutively and heated to 60 °C for one hour in an oven. The target liquids were poured into a pre-cleaned bubbler flask using a glass funnel. Subsequently, two steel tubes attached with a Teflon cork were inserted into the inlet and outlet of the bubbler (Figure S2). The steel tubes allowed nitrogen to flow through the inlet and finally to the outlet of the bubbler. The Teflon cork made the bubbler system airtight to prevent the escaping or leakage of generated target vapor. After the bubbler flask was used in the experiments, it was stored in the Chemsafe systems Storage Cabinet—Samillab-AL-D 1002 (Air clean systems, Creedmoor, NC, USA).

A QCM controller system was used to measure the sensing performance of the hybrid composites [43], as shown in Figures S3 and S5. The SAW sensor setup in the presented study was developed in a laboratory clean room. The setup consisted of the detection chamber, test board, vector network analyzer (VNA-MS46122A) (Anritsu, Richardson, TX, USA), and SMA connector, as shown in Figures S4 and S6.

Gas response measurements were carried out by positioning the hybrid-composite-coated QCM and SAW sensor inside the flow cell or detection chamber and blowing the diluted target vapor over them while concurrently monitoring the frequency changes in the sensors. All the experiments were conducted inside a clean booth, in which the temperature and humidity were regulated with the air-conditioned environment maintained at 22 ± 2 °C (LG Whisen-LPNW1451VJ, LG, Seoul, Korea). The relative humidity was 25–30% during the experiment in the QCM and SAW sensor (Fluke 971—Fluke, Everett, DC, USA, and ETP101—All-sun, Zhangzhou, China).

### 2.5. Characterization Methods

FTIR spectroscopy of the hybrid composites was conducted using the Vertex 80 v FT-IR Spectrometers (Bruker, Billerica, MA, USA). XRD of the hybrid composites was investigated using an X’pert PRO MRD (Philips/Panalytical, Malvern, UK). The surface morphologies of the hybrid composites were analyzed using the FE-SEM—S-4300SE (Hitachi, Tokyo, Japan). The morphological structure, thickness, and diameter of the hybrid composites were investigated by FE-TEM—JEM-2100F (Jeol, Akishima, Japan). X-ray photoelectron spectroscopy (XPS, K-Alpha, Thermo Fisher Scientific, Waltham, MA, USA) was used to analyze the elemental composition of the composite materials. The QCM controller system (QCM200, Stanford Research System, Sunnyvale, CA, USA) was used to measure the frequency changes during adsorption and desorption.

## 3. Results and Discussion

### 3.1. FT-IR

**Figure 3:**
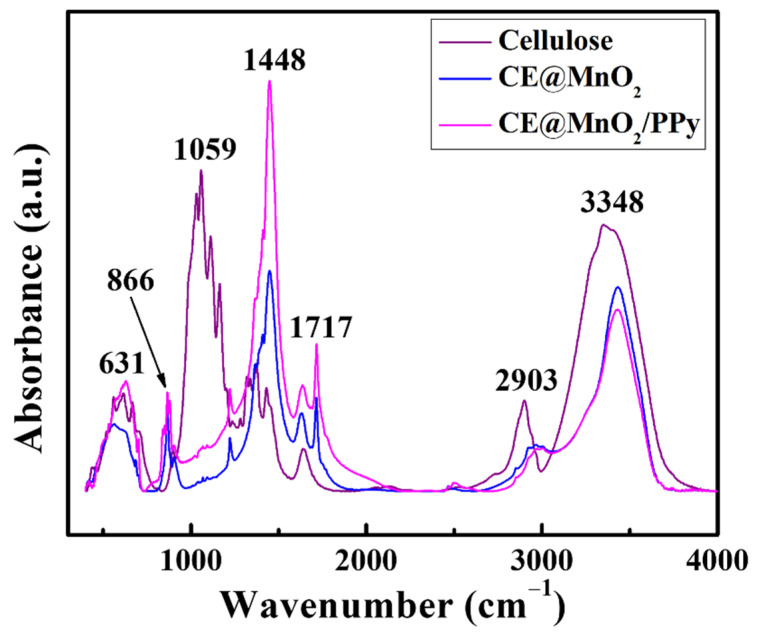
FTIR analysis of the Cellulose, MnO_2_@CE, and MnO_2_@CE/PPy.

FTIR spectroscopy is a vital tool to characterize the synthesized composites based on the absorption of the infrared spectrum. FTIR analysis of CE, MnO_2_@CE, and MnO_2_@CE/PPy was conducted to confirm the presence of polypyrrole, CE, and MnO_2_, as shown in Figure 3. CE shows a strong absorption peak at 1059, 2903, and 3348 cm^–1^. The peak at 1059 cm^–1^ indicates the C–O group from the secondary alcohols and ether function in the cellulose chain backbone [44]. The peaks at 2903 and 3348 cm^–1^ represent the stretching and deformation vibrations of the C–H group and the stretching of the hydroxyl (O–H) group, respectively. The absorption peaks between 400–800 cm^–1^ demonstrate the Mn–O lattice vibration band in this region [45]. The addition of polypyrrole is confirmed by the absorption peak at 1448 cm^–1^, which represents the C–N amide and C–H bending, which is due primarily to polymerized polypyrrole. Furthermore, polypyrrole reacts with the O–H group of cellulose [46], which resulted in a decrease in the O–H group in MnO_2_@CE/PPy, which can be observed vividly at an absorption peak of around 3348 cm^–1^. Hence, FTIR analysis confirmed the presence of polypyrrole, CE, and MnO_2_ in MnO_2_@CE/PPy.

### 3.2. XRD Analysis

**Figure 4:**
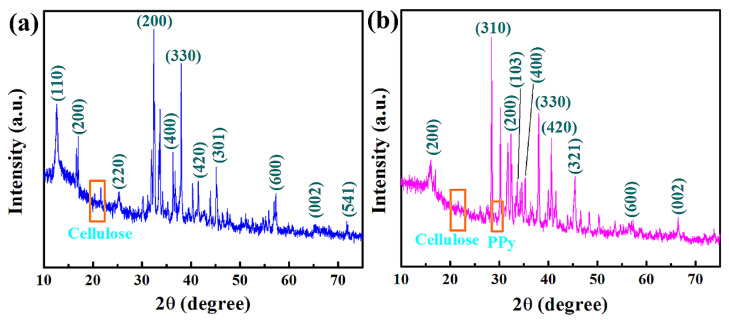
XRD analysis of (**a**) MnO_2_@CE and (**b**) MnO_2_@CE/PPy.

XRD is a tool to characterize crystallinity and crystallite sizes. Figure S7 shows the peaks at 14.9° and 22.4° 2θ, which were assigned to the 101 and 002 planes, respectively. This indicates the crystalline structure of cellulose, which has a crystallinity range of 64–91%. The XRD results (see Figure 4a) revealed a polycrystalline tetragonal structure of α–MnO_2_. The XRD peaks at 12.6°, 17.0°, 25.4°, 32.4°, 33.7°, 36.3°, 38.0°, 41.5°, 43.9°, 45.1°, 57.3°, 65.3°, and 71.7° 2θ correspond to 110, 200, 220, 200, 103, 400, 330, 420, 301, 321, 600, 002, and 541 planes, respectively, that exhibit the tetragonal structure of MnO_2_ in MnO_2_@CE composites (JCPDS: 44-0141). The peaks at 20.3° and 21.6° 2θ revealed the presence of cellulose in MnO_2_@CE composites. Figure 4b shows the XRD characteristics peaks of MnO_2_@CE/PPy. In addition, the XRD peaks at 16.0°, 28.4°, 32.4°, 33.7°, 35.3°, 38.0°, 40.6°, 45.4°, 57.3°, and 66.5° 2θ correspond to the 200, 310, 200, 103, 400, 330, 420, 321, 600, and 002 planes, respectively, that reveal the tetragonal structure of α—MnO_2_ (JCPDS: 44-0141 and 24-0734) [47]. The observed XRD peak at 29.3° 2θ might have arisen from the addition of polypyrrole [48] in the MnO_2_@CE/PPy composites. The low-intensity peaks at 21.6° 2θ were revealed by the cellulose in MnO_2_@CE/PPy, in which the intensity is slightly diminished compared to MnO_2_@CE/PPy and MnO_2_@CE composites. Hence, polypyrrole, cellulose, and manganese oxide are included in the MnO_2_@CE/PPy composites.

### 3.3. XPS Analysis

**Figure 5:**
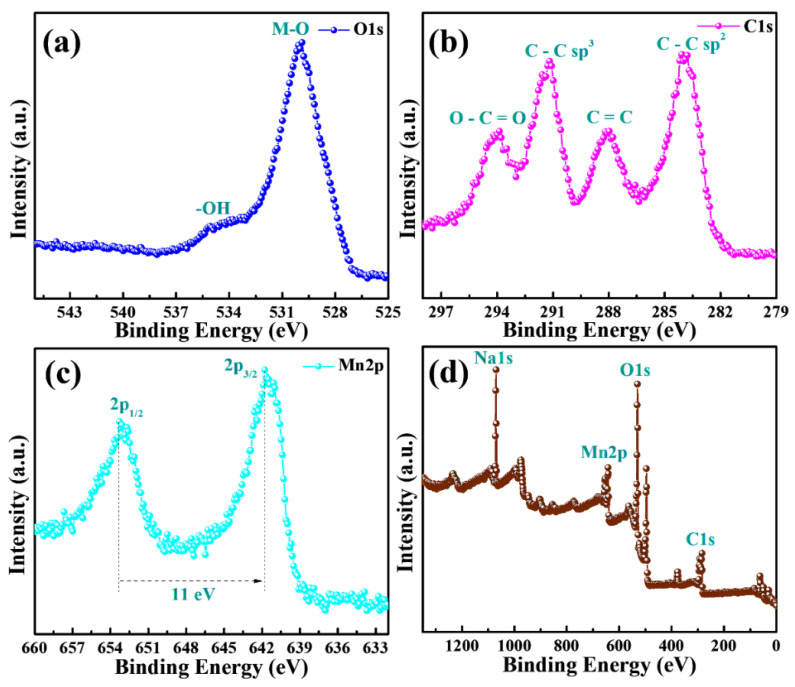
XPS results of MnO_2_@CE_._ (**a**) O 1 s, (**b**) C 1 s, and (**c**) Mn 2*p* binding energy and (**d**) survey spectrum.

XPS was used to characterize the elemental composition of the synthesized MnO_2_@CE and MnO_2_@CE/PPy composites. Figure 5a–d show the XPS characteristics peak with respect to the element binding energy for the MnO_2_@CE composites. Figure 5a shows the O 1 s element peaks at 530 and 535 eV, corresponding to the metal–oxygen bonding and hydroxyl (O–H) group, respectively, in the MnO_2_@CE composites. Figure 5b depicts the C 1 s element peaks at the binding energies of 284, 288, 291, and 294 eV, which corresponds to the C–C *sp*^2^, C=C, C–C *sp*^3^, and O–C=O, respectively. The Mn 2*p* characteristic peaks are shown in Figure 5c at 642 and 653 eV, corresponding to 2*p*_3/2_ and 2*p*_1/2_ with an energy difference of 11 eV [49]. The survey spectrum (Figure 5d) revealed the presence of O, C, Mn, and Na in the MnO_2_@CE composites.

**Figure 6:**
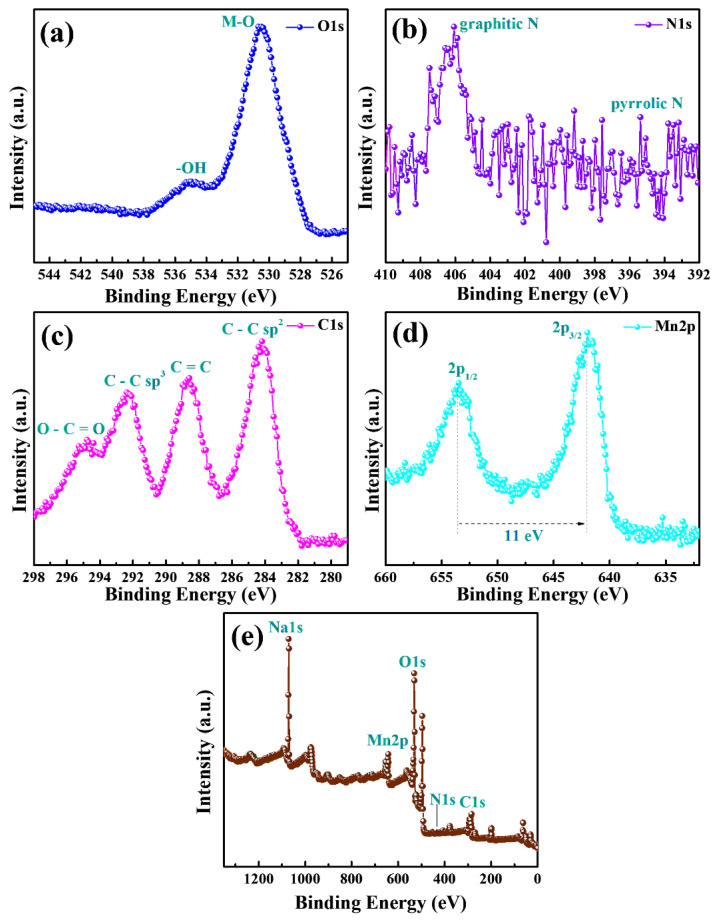
XPS analysis of MnO_2_@CE/PPy. (**a**) O 1 s, (**b**) N 1 s, (**c**) C 1 s, and (**d**) Mn 2*p* binding energy and (**e**) survey spectrum.

Figure 6a–e show the XPS characteristic peaks and survey spectrum of MnO_2_@CE/PPy composites. Figure 6a exhibits the O 1 s element peaks at 530 and 535 eV, corresponding to the metal–oxygen bond and O–H group, respectively, in MnO_2_@CE/PPy composites. Figure 6b shows the N 1 s characteristic peaks at the binding energies of 395 and 406 eV, corresponding to the pyrrole N and graphitic N, respectively [50]. Figure 6c presents the C 1 s element peaks at the binding energies of 284, 289, 292, and 295 eV, which correspond to the C–C *sp*^2^, C=C, C–C *sp*^3^, and O–C=O, respectively. Figure 6d shows the Mn 2*p* characteristic peaks at 642 and 653 eV, corresponding to the 2*p*_3/2_ and 2*p*_1/2_, with an energy difference of 11 eV [49]. The survey spectrum (Figure 6e) reveals the presence of O, C, N, Na, and Mn in the MnO_2_@CE composites.

### 3.4. SEM

**Figure 7:**
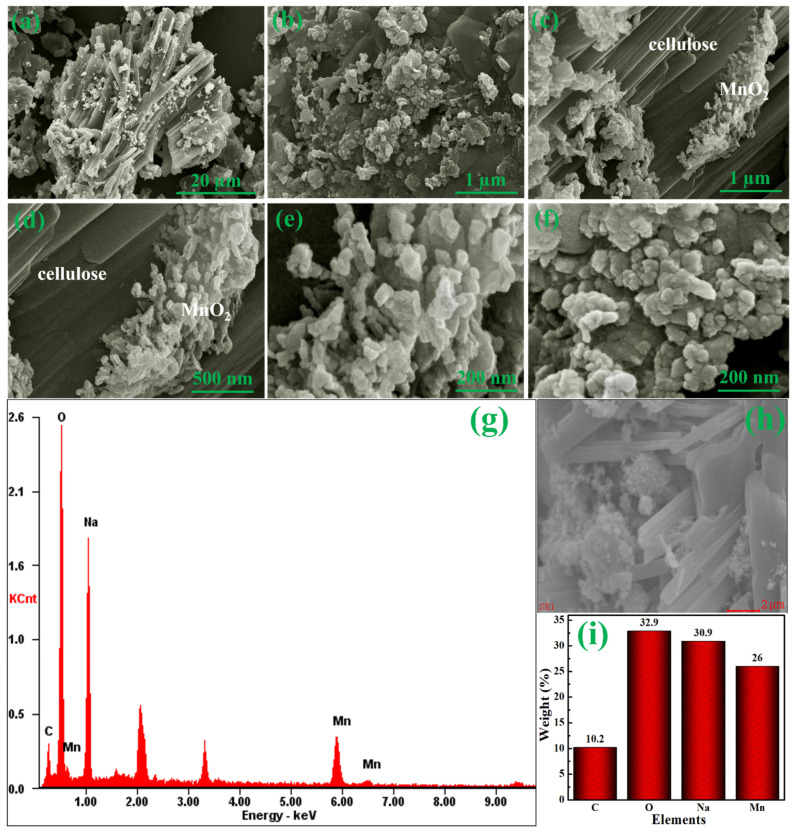
FE-SEM images of MnO_2_@CE at the magnification of (**a**) 20 µm, (**b**) 1 µm, (**c**) 1 µm, (**d**) 500 nm, (**e**) 200 nm, (**f**) 200 nm, and (**g**–**i**) EDAX profile with elemental percentage by weight.

The surface morphologies of the CE and synthesized composites were investigated by the FE-SEM. Figure S8a–f show the FE-SEM morphology of CE at different magnifications. The nanosheet-like structure is stacked on top of each other. The cellulose microcrystals agglomerate together, forming an amorphous shape [51]. Figure 7a–f depict the FE-SEM morphology of MnO_2_@CE, which resembles the shape of clustered sea crystals. The extended stack in MnO_2_@CE composites (Figure 7a,c,d) emerged from the addition of CE, while manganese oxide was responsible for the non-uniform large semispherical disc-like structure [52] with sizes ranging from 50–300 nm. The non-uniform semispherical discs were agglomerated and stacked on top of each other, forming a hummock-like structure. Figure 7g–i illustrate the EDAX profile of MnO_2_@CE, which confirms the presence of Mn, O, Na, and C. The elemental percentages by weight of Mn, O, Na, and C were 26.0%, 32.9%, 30.9%, and 10.2%, respectively. This is convincing evidence for the inclusion of MnO_2_ in the hybrid composite of MnO_2_@CE.

**Figure 8:**
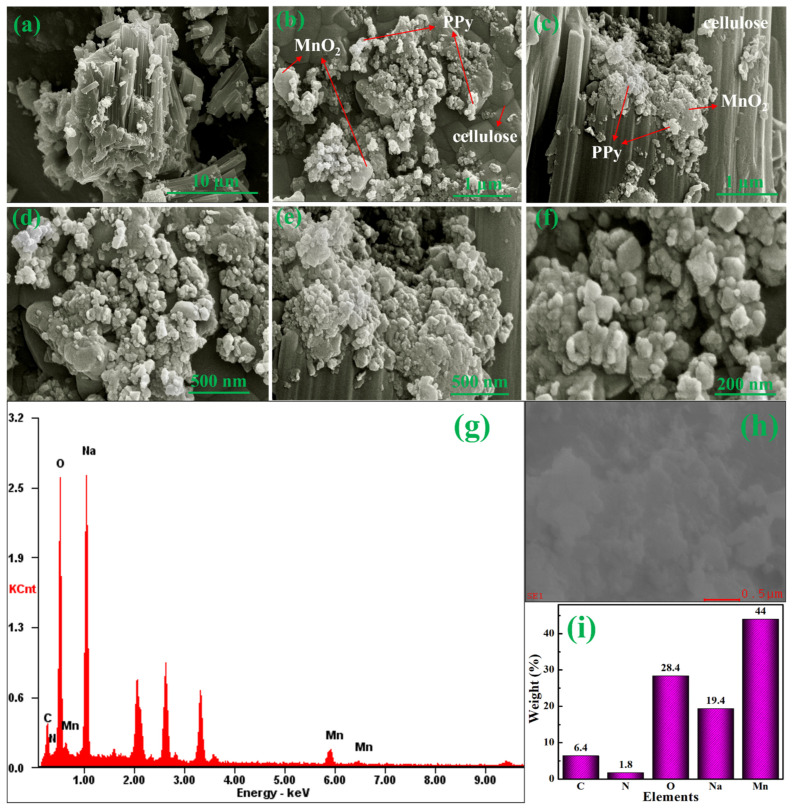
FE-SEM images of MnO_2_@CE/PPy at the magnification of (**a**) 10 µm, (**b**) 1 µm, (**c**) 1 µm, (**d**) 500 nm, (**e**) 500 nm, (**f**) 200 nm, and (**g**–**i**) EDAX profile with elemental percentage by weight.

In addition, Figure 8a–f show the FE-SEM images of MnO_2_@CE/PPy at different magnifications. Polypyrrole was decorated on the top of the stacked MnO_2_ and CE, which provided more surface area for adsorption and desorption kinetics [53]. The polypyrrole was responsible for inhomogeneous small semispherical grain-like structures [52] with a size of 15–30 nm. Figure 8g–i illustrate the EDAX profile and elemental percentage by the weight of MnO_2_@CE/PPy, which confirmed the presence of Mn, O, Na, N, and C. The elemental percentages by weight of Mn, O, Na, N, and C were 44.0%, 28.4%, 19.4%, 1.8%, and 6.4%, respectively. Hence, MnO_2_, CE, and PPy were present in the MnO_2_@CE/PPy composites.

### 3.5. TEM

**Figure 9:**
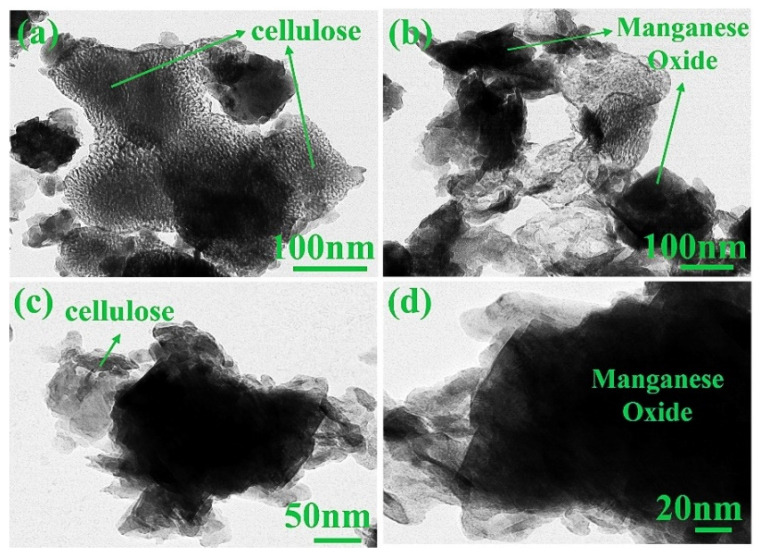
FE-TEM images of MnO_2_@CE at the magnification of (**a**) 100 nm, (**b**) 100 nm, (**c**) 50 nm, and (**d**) 20 nm.

**Figure 10:**
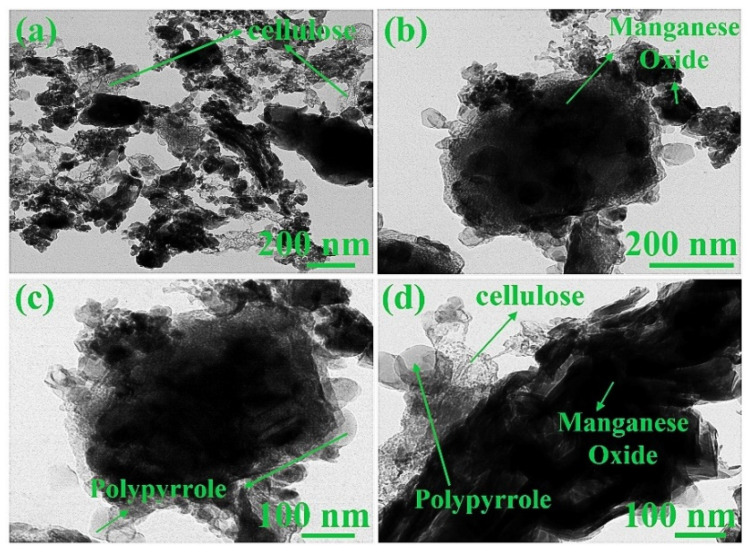
FE-TEM images of MnO_2_@CE/PPy at the magnification of (**a**) 200 nm, (**b**) 200 nm, (**c**) 100 nm, and (**d**) 100 nm.

FE-TEM was used to characterize the CE and synthesized composites at different magnifications. Figure S9a–f show FE-TEM images of cellulose under different magnifications. CE has a small grain-like structure in a spherical shape [54]. The cellulose morphology and its shape differ significantly upon the preparation method and its precursor [55]. The images (Figure S9c,e,f) also suggest that the chain bundles are together randomly. However, the bonding of chains is restricted to short lengths [51]. Figure 9a–d show the FE-TEM images of MnO_2_@CE composites at various magnifications. The jelly-like structure was attributed to the addition of cellulose. The black amorphous shape was attributed to the manganese oxide in the MnO_2_@CE composites [56]. Figure 10a–d present the FE-TEM analysis of the MnO_2_@CE/PPy. The inclusion of polypyrrole is clearly confirmed due to the surface modification onto the MnO_2_@CE matrix in MnO_2_@CE/PPy composites [50]. The nano-disc-like structure or grains (15–30 nm) come from the polypyrrole, which was decorated over the surface of hybrid composites. Hence, MnO_2_@CE/PPy is a composite of manganese oxide, cellulose, and polypyrrole within its matrix.

### 3.6. Experimentation in the QCM Sensor

**Figure 11:**
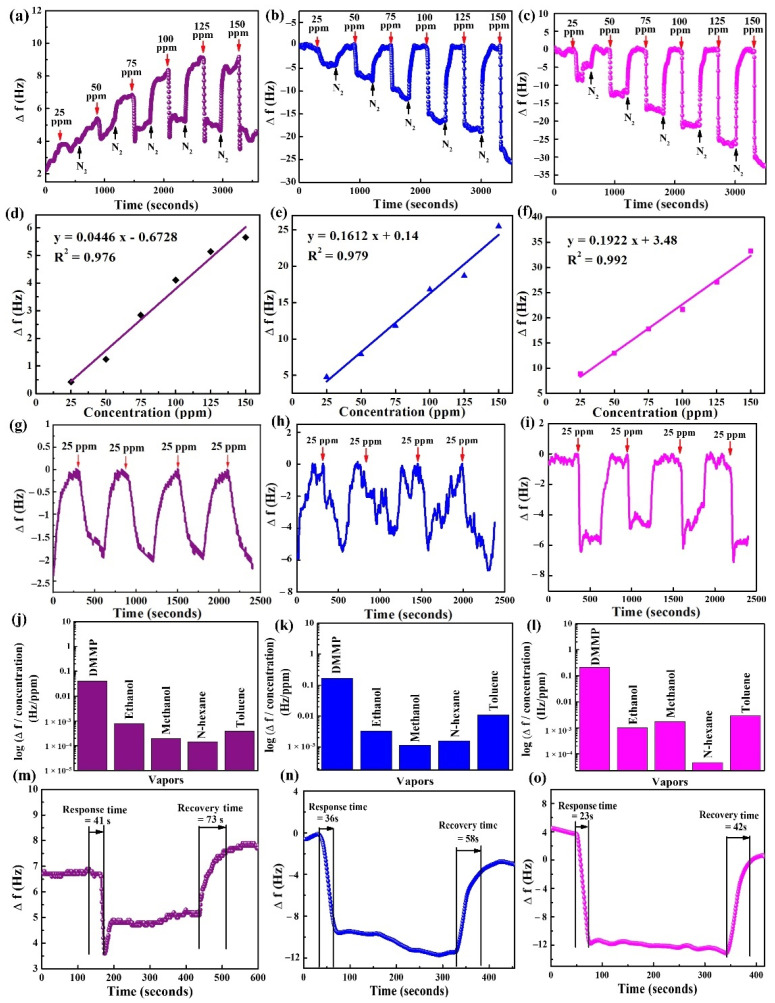
Frequency shifts of (**a**) CE, (**b**) MnO_2_@CE, (**c**) MnO_2_@CE/PPy; linearity of (**d**) CE, (**e**) MnO_2_@CE, (**f**) MnO_2_@CE/PPy; Repeatability of (**g**) CE, (**h**) MnO_2_@CE, (**i**) MnO_2_@CE/PPy, for 25 ppm DMMP; selectivity of (**j**) CE, (**k**) MnO_2_@CE, (**l**) MnO_2_@CE/PPy; and response/recovery times of (**m**) CE, (**n**) MnO_2_@CE, (**o**) MnO_2_@CE/PPy, for 75 ppm DMMP vapor; in the QCM sensor.

The frequency responses of the cellulose- and hybrid-composite-coated QCM sensors were investigated to detect DMMP vapor varying from 25 to 150 ppm, illustrated in Figure 11a–c. The DMMP vapor was exposed for five minutes, followed by five minutes of purging gas. For the DMMP concentration of 25 ppm, the frequency shifts (Δ*f*) of CE, MnO_2_@CE, and MnO_2_@CE/PPy were 0.4, 4.8, and 8.9 Hz, respectively. In addition, for 150 ppm of DMMP, CE, MnO_2_@CE, and MnO_2_@CE/PPy showed the Δ*f* of 5.6, 25.5, and 33.3 Hz, respectively. The frequency shift of CE shows an upward trend, which may be attributed to possible mass loss due to the repeated adsorption and desorption process. Hence, it may have caused the volumetric changes, allowing a small portion of the sensing material to fall off the CE surface [57]. The fast recovery of the QCM sensor when the QCM sensor was purged with nitrogen showed that the interaction between the sensing materials and DMMP was the weak force of attraction or hydrogen bonding [19,58].

Figure 11d–f show the linear behavior of the QCM sensor coated with CE, MnO_2_@CE, and MnO_2_@CE/PPy in response to the DMMP concentration from 25–150 ppm. CE, MnO_2_@CE, and MnO_2_@CE/PPy presented with COD (R^2^) of 0.976, 0.979, and 0.992, respectively. The sensing materials displayed excellent R^2^, which may be because of good cohesive contact between the electrodes and sensing materials. In addition, the practical applicability of the composite materials is ensured [59].

Figure 11g–i illustrate the reproducibility of the QCM sensor coated with CE, MnO_2_@CE, and MnO_2_@CE/PPy, respectively, for 25 ppm DMMP vapor. CE showed an average Δ*f* of 2.08 Hz with a standard deviation (SD) of 0.09 Hz, while the average Δ*f* of MnO_2_@CE was 5.52 Hz with a SD of 0.73 Hz, and the average Δ*f* of MnO_2_@CE/PPy was 6.15 Hz with a SD of 0.79 Hz. From the figures, small drifts can be seen during the detection process, primarily due to the concentration differences, as the dilution process cannot control the vapor concentration in a highly accurate way [60].

CE, MnO_2_@CE, and MnO_2_@CE/PPy were exposed to the DMMP and several volatile organic compounds (VOCs), including ethanol, methanol, n-hexane, and toluene, at a constant flow rate of 200 sccm. Figure 11j–l show the logarithmic frequency shift per concentration of CE, MnO_2_@CE, and MnO_2_@CE/PPy. Despite the higher concentrations of VOCs compared to DMMP, the synthesized materials showed a relatively higher frequency shift towards the DMMP. Therefore, it can detect DMMP among other VOCs at higher concentrations.

Figure 11m–o display the response and recovery times of CE, MnO_2_@CE, and MnO_2_@CE/PPy, respectively, in detecting 75 ppm DMMP. MnO_2_@CE/PPy showed the shortest response (~23 s) and recovery times (~42 s). In addition, CE and MnO_2_@CE showed response times of ~41 s and ~36 s, respectively, and displayed recovery times of ~73 s and ~58 s, respectively. The recovery times are two times longer than the response times for the pristine CE and its hybrid composite materials. Furthermore, the repeated adsorption and desorption phenomena indicate that the DMMP is attached to the surface of sensing materials with a weak force of attraction or Van der Waals force [19,61].

Finally, the QCM sensor test was used for the DMMP concentration ranging from 25–150 ppm at room temperature. The QCM test demonstrated that the frequency shifts of MnO_2_@CE and MnO_2_@CE/PPy are ~12 and ~22.3 times higher than that of pristine cellulose at 25 ppm DMMP, respectively, in the QCM sensor. At 150 ppm, the frequency shifts were ~4.6 and ~6 times higher than the pristine cellulose. The hybrid composites of the MnO_2_@CE/PPy showed an excellent R^2^ of 0.992 in the detection of DMMP from 25 to 150 ppm. The MnO_2_@CE/PPy showed excellent repeatability while detecting 25 ppm DMMP, suggesting that the DMMP may be attached to the surface of the sensing materials through hydrogen bonding [61]. Despite the higher concentration of the VOCs compared to the DMMP, the sensing materials still managed to detect the DMMP. Finally, MnO_2_@CE/PPy showed the shortest response and recovery times of ~23 s and ~42 s, respectively, at 75 ppm DMMP in the QCM sensor.

### 3.7. Experimentation in the SAW Sensor

**Figure 12:**
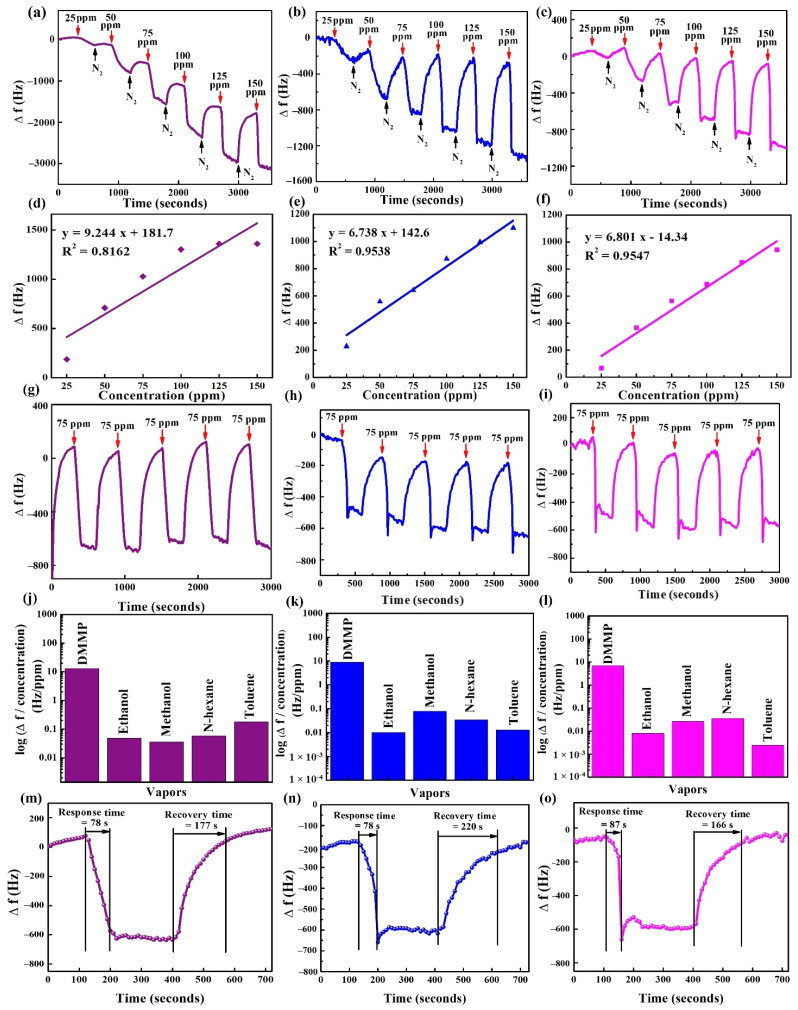
Frequency shifts of (**a**) CE, (**b**) MnO_2_@CE, (**c**) MnO_2_@CE/PPy; linearity of (**d**) CE, (**e**) MnO_2_@CE, (**f**) MnO_2_@CE/PPy; Repeatability of (**g**) CE, (**h**) MnO_2_@CE, (**i**) MnO_2_@CE/PPy, for 75 ppm DMMP; selectivity of (**j**) CE, (**k**) MnO_2_@CE, (**l**) MnO_2_@CE/PPy; and response/recovery times of (**m**) CE, (**n**) MnO_2_@CE, (**o**) MnO_2_@CE/PPy, for 75 ppm DMMP; in the SAW sensor.

Figure 12a–c present the real-time detection of the frequency responses in the SAW sensors coated with CE, MnO_2_@CE, and MnO_2_@CE/PPy for the detection of 25–150 ppm DMMP vapor, respectively. For a concentration of 25 ppm, CE, MnO_2_@CE, and MnO_2_@CE/PPy presented with frequency responses of 187 Hz, 276 Hz, and 78 Hz, respectively. CE, MnO_2_@CE, and MnO_2_@CE/PPy exhibited frequency responses of 1360 Hz, 1104 Hz, and 943 Hz, respectively, for a DMMP concentration of 150 ppm. The observed characteristics can be elucidated by the interaction of the hydroxyl group of cellulose with the amine group of the polypyrrole, which tends to decrease the number of hydroxyl groups used in hydrogen bonding when detecting DMMP vapor [46,62].

CE-, MnO_2_@CE-, and MnO_2_@CE/PPy-coated SAW sensors were exposed to the DMMP varying from 25–150 ppm to investigate the linearity, as shown in Figure 12d–f, respectively. CE, MnO_2_@CE, and MnO_2_@CE/PPy showed R^2^ = 0.8162, 0.9538, and 0.9547, respectively. Compared to the CE, the composite materials demonstrated an increase in R^2^, which may result from interactions between the electrode and sensing materials [2].

CE-, MnO_2_@CE-, and MnO_2_@CE/PPy-coated SAW sensors were exposed to 75 ppm DMMP to investigate the reproducibility, as shown in Figure 12g–i, respectively. CE showed an average Δ*f* of 757 Hz with a SD of 25 Hz, while the average Δ*f* of MnO_2_@CE was 508 Hz with a SD of 32 Hz, and the average Δ*f* of MnO_2_@CE/PPy was 638 Hz with a SD of 30 Hz. Because there were no significant changes in the response of the CE and composite-material-coated SAW sensor, it possesses excellent reproducibility [19,63,64].

CE-, MnO_2_@CE-, and MnO_2_@CE/PPy-coated SAW sensors were exposed to the DMMP and several VOCs, such as n-hexane, methanol, toluene, and ethanol, at the constant flow rate of 100 sccm. These sensing materials are sensitive to DMMP rather than potential interferences (Figure 12j–l). For example, the n-hexane vapor concentration was 21,915.27 ppm, which is 219-fold greater than DMMP. On the other hand, the frequency response of CE, MnO_2_@CE, and MnO_2_@CE/PPy were more than 225-, 254-, and 192-fold greater in DMMP than in n-hexane [59]. Hence, these materials can be used as potential candidates for DMMP sensing.

Figure 12m–o depict the response and recovery times of SAW sensors coated with CE, MnO_2_@CE, and MnO_2_@CE/PPy, respectively. CE and MnO_2_@CE showed the shortest response time of ~78 s, while MnO_2_@CE/PPy presented with an ~87 s response time. On the other hand, the shortest recovery time (~166 s) was shown by MnO_2_@CE/PPy followed by the CE and MnO_2_@CE with ~177 s and ~220 s, respectively. The recovery times were ~two–three times longer than response times. The longer recovery times in SAW and QCM sensors may have resulted from the low desorption rate of attached DMMP molecules from the surface of the coated materials [65].

A SAW sensors test for the DMMP concentrations ranging from 25–150 ppm was performed at room temperature. The SAW sensors test demonstrated the frequency shifts of 187 Hz, 276 Hz, and 78 Hz for CE, MnO_2_@CE, and MnO_2_@CE/PPy, respectively, at 25 ppm DMMP. The hydrogen bonding between the cellulose and poly pyrrole [66] may have caused the decrease in the frequency shift of the SAW sensor coated with MnO_2_@CE/PPy. CE, MnO_2_@CE, and MnO_2_@CE/PPy presented with R^2^ = 0.8162, 0.9538, and 0.9547, respectively, in detection of DMMP from 25–150 ppm. The pristine CE and its hybrid composite have shown excellent repeatability while detecting 75 ppm DMMP, suggesting that the DMMP might be attached to the surface of the sensing materials by the formation of hydrogen bonding or van der Waals force [61]. Furthermore, the SAW sensor showed that the pristine CE and its hybrid composite showed higher frequency shifts towards the DMMP rather than potential interferences. For 75 ppm DMMP, the response times of MnO_2_@CE and MnO_2_@CE/PPy were 1.0 and ~1.1 times longer than that of pristine cellulose, respectively. In addition, the recovery time was ~1.2 and ~0.9 times longer than the pristine cellulose. Therefore, the sensing performance of the hybrid composites was also confirmed by experimentation with the SAW sensor.

### 3.8. Sensing Mechanism

**Figure 13:**
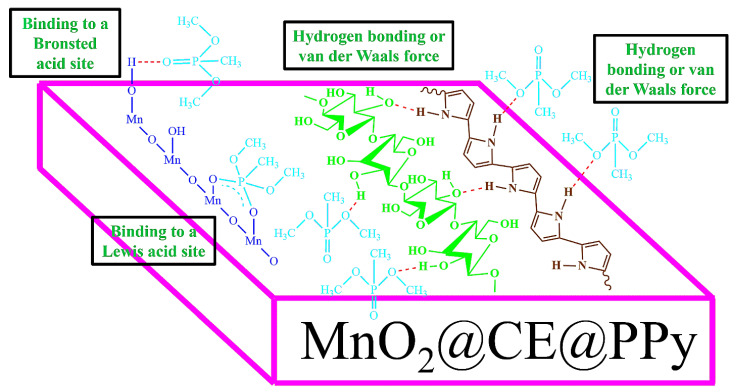
Proposed sensing mechanism of MnO_2_@CE/PPy with the DMMP. The figure is not to scale.

The MnO_2_ surface may react with the DMMP vapor by bonding with a Lewis acid site, giving rise to a bidentate structure by attaching the metal site with the nearest O–H group, as well as the Bronsted acid site [20]. The amine group of polypyrrole reacts with the hydroxyl group of the cellulose to form the CE–PPy composites [46]. Therefore, the hydroxyl group of cellulose reacts with O═P of DMMP via hydrogen bonding [67] or Van der Waals force, see (Figure 13). Furthermore, the formation of hydrogen bonds could occur between the electron-rich O atoms in the P═O bond of DMMP and electron-deficient H atoms in the N–H bond of PPy [27,66]. Because DMMP is a strong electron donor, when reacting with PPy, it leads to a decrease in the number of holes in PPy and increases its electrical resistance while bonding with the hydrogen bond simultaneously [68]. On the other hand, it tends to decrease the number of hydroxyl groups that combine with DMMP due to hydrogen bonding between CE and PPy, resulting in a lower frequency shift.

Cellulose has limiting properties, such as resistance to the chemical, high stiffness and strength, and poor solubility and processability in the field of sensors. Adding a small amount of metal atoms or “doping process” could enhance the properties and performance of cellulose. On the other hand, reports on the detection of CWAs simulant by the hybrid composites of cellulose and MnO_2_ are still rare. Yue et al. reported the use of ZnO-cellulose/MnO_2_ for efficient and rapid separation and treatment of oily wastewater [35]. Cellulose/*f*-CNT/MnO_2_ showed a high capacitance of 1812 mFcm^−2^, and maximum energy and power density of 251.66 µWhcm^–2^ and 24.85 mWcm^–2^, respectively, demonstrating the excellent potential for supercapacitor applications [36]. Tran et al. utilized the composites of MnO_2_ and cellulose for lithium-ion batteries that demonstrated a high discharge capacity of 305 mAhg^−1^ after 1000 cycles [37]. The synergetic effect of cellulose nanocrystal and MnO_2_ hybrid showed great potential for dye wastewater treatment, in which equilibrium decolorization of 114.5 mg/g could be reached [69]. To the authors’ knowledge, however, there are no reports on incorporating MnO_2_ with the cellulose and conducting polymer PPy to detect CWA simulant at room temperature. Hence, a comparison of synthesized MnO_2_@CE and MnO_2_@CE/PPy with its pristine cellulose to detect the CWA simulant at concentrations of 25–150 ppm was needed. In addition, the possible sensing mechanism between the DMMP and MnO_2_@CE/PPy were suggested.

## 4. Conclusions

Hybrid nanostructured composites were synthesized using pristine cellulose as a base material and tested to analyze the sensing performance to detect a simulant of G-series nerve agents at 22 ± 2 °C and relative humidity of 25–30%. CE, MnO_2_@CE, and MnO_2_@CE/PPy were characterized by FTIR spectroscopy, XRD, XPS, SEM, and TEM to examine the elemental compositions, bonds and their respective planes, energy differences, and morphological structures. FTIR spectroscopy demonstrated the absorption peaks of O–Mn–O at 631 cm^–1^, *β*-glycosidic linkage of cellulose at 866–898 cm^–1^, and polypyrrole inclusion at 1448 cm^–1^. XRD revealed the polycrystalline tetragonal structure of α-MnO_2_ in the synthesized hybrid composites. XPS showed that the characteristic peak of Mn 2*p*, which corresponds to 2*p*_3/2_ and 2*p*_1/2_, showed an energy difference of 11 eV. SEM revealed the clustered sea crystal-like structure of MnO_2_@CE and polypyrrole decorated on the surface of the MnO_2_@CE. TEM proved the inclusion of cellulose and manganese oxide in the hybrid composites. The pristine cellulose and its hybrid composites were tested in a QCM and SAW sensor to detect the frequency shift, linearity, reproducibility, selectivity, and response/recovery times for DMMP, ranging from 25 to 150 ppm. The synthesized hybrid composites showed an increased frequency shift compared to the pristine cellulose in the QCM sensor. MnO_2_@CE/PPy showed excellent R^2^ in both QCM and SAW sensors compared to CE and MnO_2_@CE. The selectivity test indicated that the CE and its hybrid composites showed an increased frequency shift to DMMP despite the higher VOC concentrations. MnO_2_@CE/PPy showed the shortest response and recovery times in the QCM sensor. The reproducibility study showed that DMMP was attached to the hydroxyl group of the cellulose and its composite via hydrogen bonding or Van der Waals forces.

## Data Availability

Not applicable.

## Supplementary Materials

The following supporting information can be downloaded at: https://www.mdpi.com/article/10.3390/ma15207313/s1, The supporting information includes the chemical structure of DMMP and CWAs (Figure S1), vapor generating bubbler and analyte delivery system (Figure S2), schematic diagram of the gas sensing system in the QCM and its setup (Figures S3 and S5), schematic diagram of the gas sensing system in the SAW sensor and its setup (Figures S4 and S6), XRD (Figure S7), FE-SEM (Figure S8), and FE-TEM (Figure S9) of microcrystalline cellulose.
