# Supplementary material for: Synthesis and Characterization of MnO2@Cellulose and Polypyrrole-Decorated MnO2@Cellulose for the Detection of Chemical Warfare Agent Simulant
Source: Materials (Basel). 2022 Oct 19;15(20):7313. doi: 10.3390/ma15207313 (PMC9606964; doi:10.3390/ma15207313)
Supplement: Supplementary file 1 [file materials-15-07313-s001.zip › materials-1926692-supplementary.pdf]

Supplementary Information

Clustered sea crystals like structure of MnO_2@Cellulose and polypyrrole-decorated MnO_2@Cellulose nanostructured composite materials for the detection of Chemical Warfare Agent simulant

Sanjeeb Lama [1], Sumita Subedi [2], Sivalingam Ramesh [3], Kyeongho Shin [1], Young-Jun Lee [1]*, Joo-Hyung Kim [1]*

[1] *Laboratory of Intelligent Devices and Thermal Control, Department of Mechanical Engineering, Inha University, Incheon 22212, Korea*

[2] *Department of chemistry and chemical engineering, Inha University, Incheon 22212, Korea*

[3] *Department of Mechanical, Robotics, and Energy Engineering, Dongguk University-Seoul, Seoul 04620, Korea*

* *Corresponding author: Joo-Hyung Kim: joohyung.kim@inha.ac.kr*

FIGURES:

Figure S1. Chemical structure of (a) DMMP (b) Sarin, (c) Soman, and (d) Tabun. The figure is not to scale. 3

Figure S2. Bubbler flask used in the presented study (b) Schematic diagram of the vapor generating process in the bubbler. 4

Figure S3. Schematic diagram of the gas sensing system for QCM sensor. 5

Figure S4. Schematic diagram of the gas sensing system for SAW sensor. 6

Figure S5. (a) QCM200 setup (digital controller, QCM crystals in Petri dish, crystal controller, and flow cell) (b) QCM flow cell (c) QCM signal monitoring program (d) Schematic of gas flow pattern in the flow cell. 7

Figure S6. (a) SAW sensor setup for the experimentation (b) Top view of the SAW sensor mounted on the test board (c) Front view of detection chamber with top cover for gas monitoring (d) Schematic of the gas flow diagram in the detection chamber (e) SAW sensor monitoring program to check signals from SAW ports. 8

Figure S7. XRD analysis of cellulose. 9

Figure S8. FE-SEM images of cellulose under different magnifications. 10

Figure S9. FE-TEM images of cellulose under different magnifications. 11

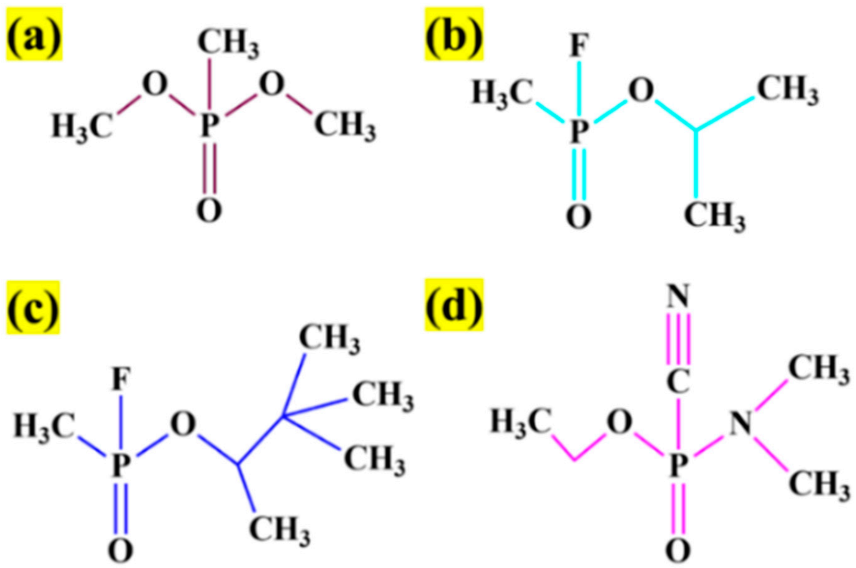

Figure S1. Chemical structure of (a) DMMP (b) Sarin, (c) Soman, and (d) Tabun. The figure is not to scale.

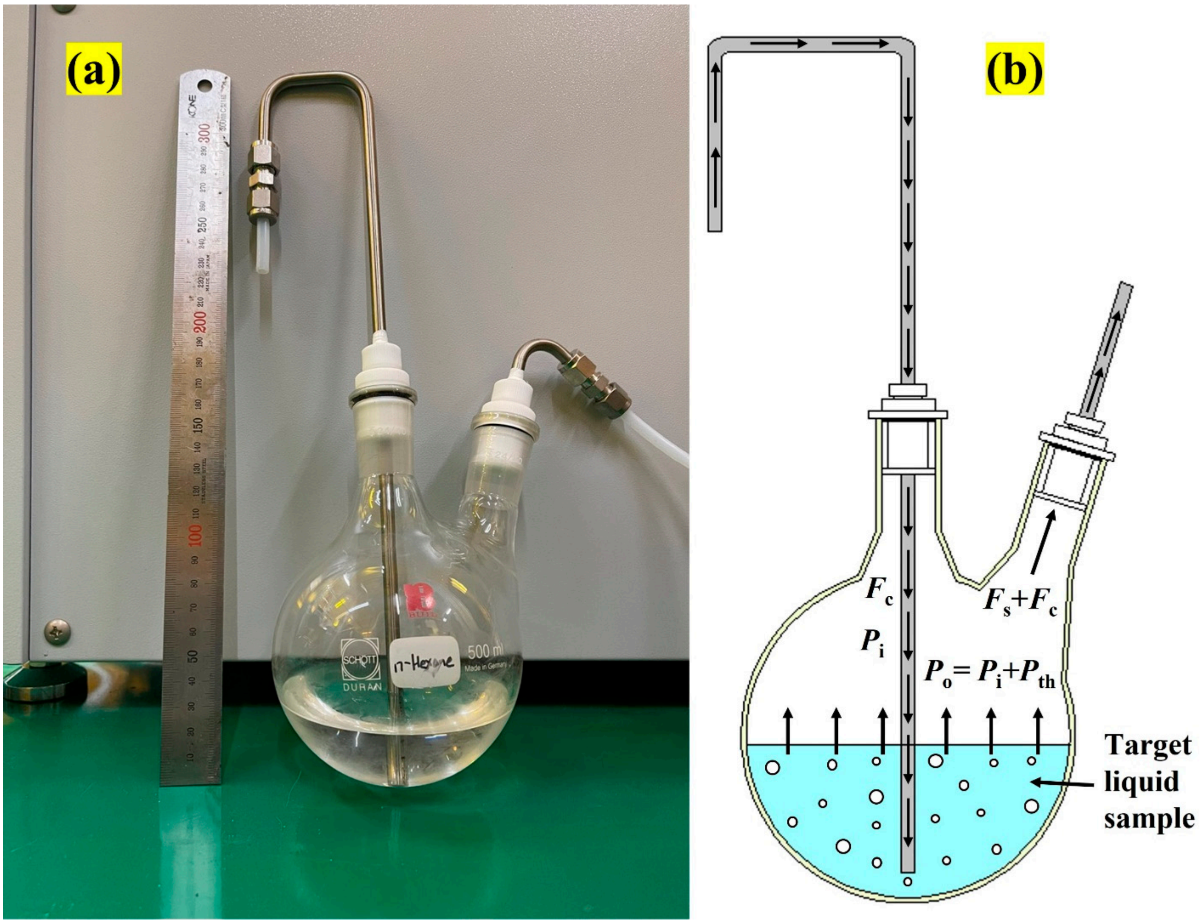

Figure S2. Bubbler flask used in the present study (b) Schematic diagram of the vapor generating process in the bubbler.

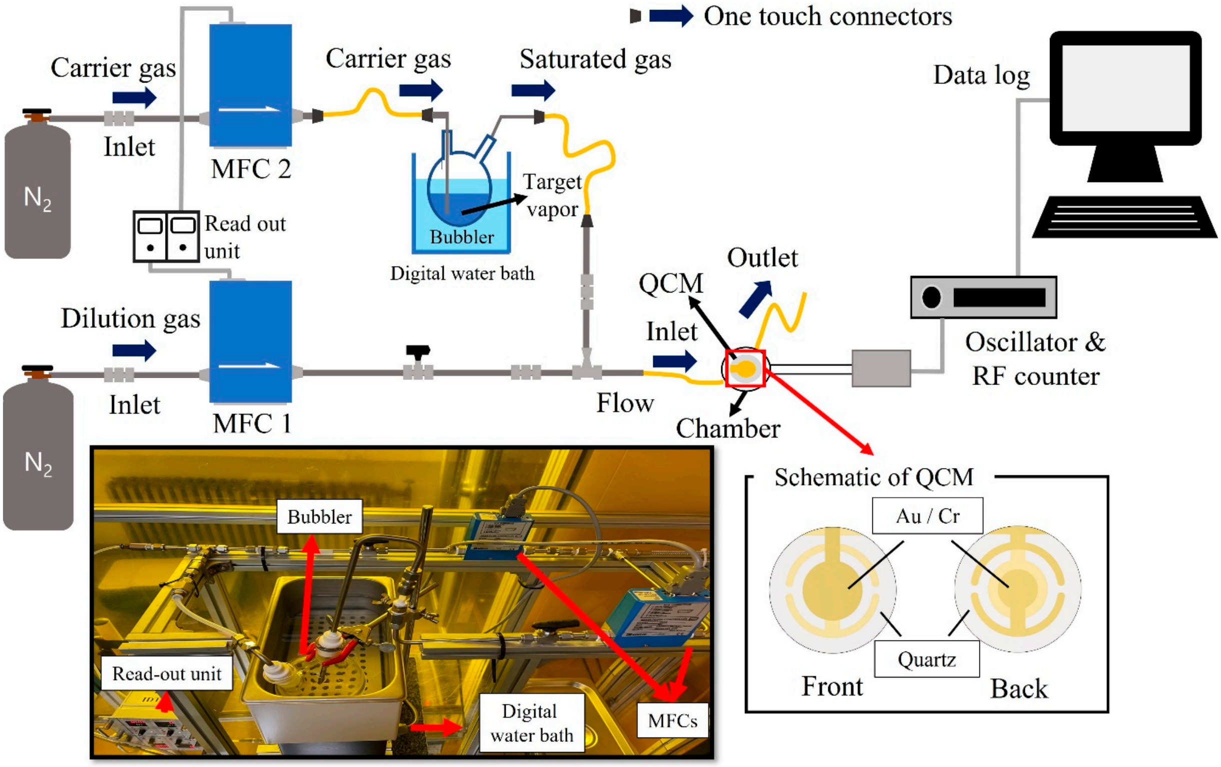

Figure S3. Schematic diagram of a gas sensing system for the QCM sensor.

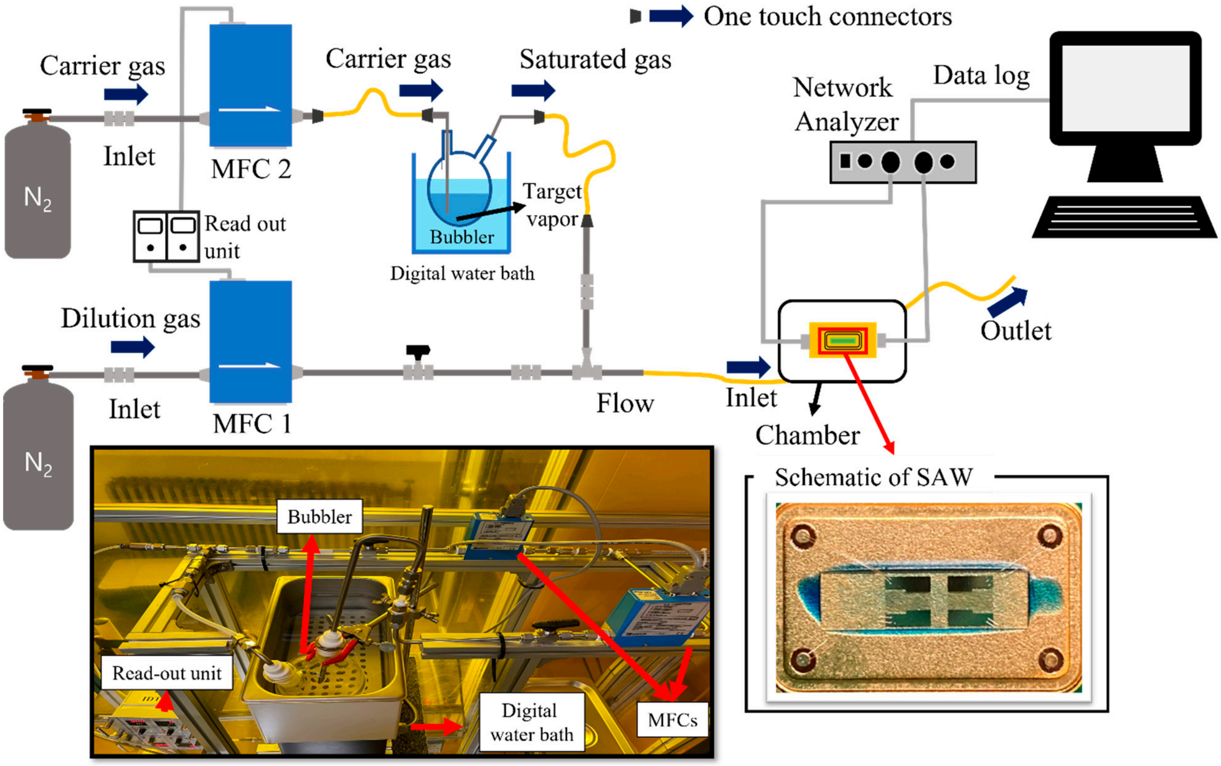

Figure S4. Schematic diagram of a gas sensing system for the SAW sensor.

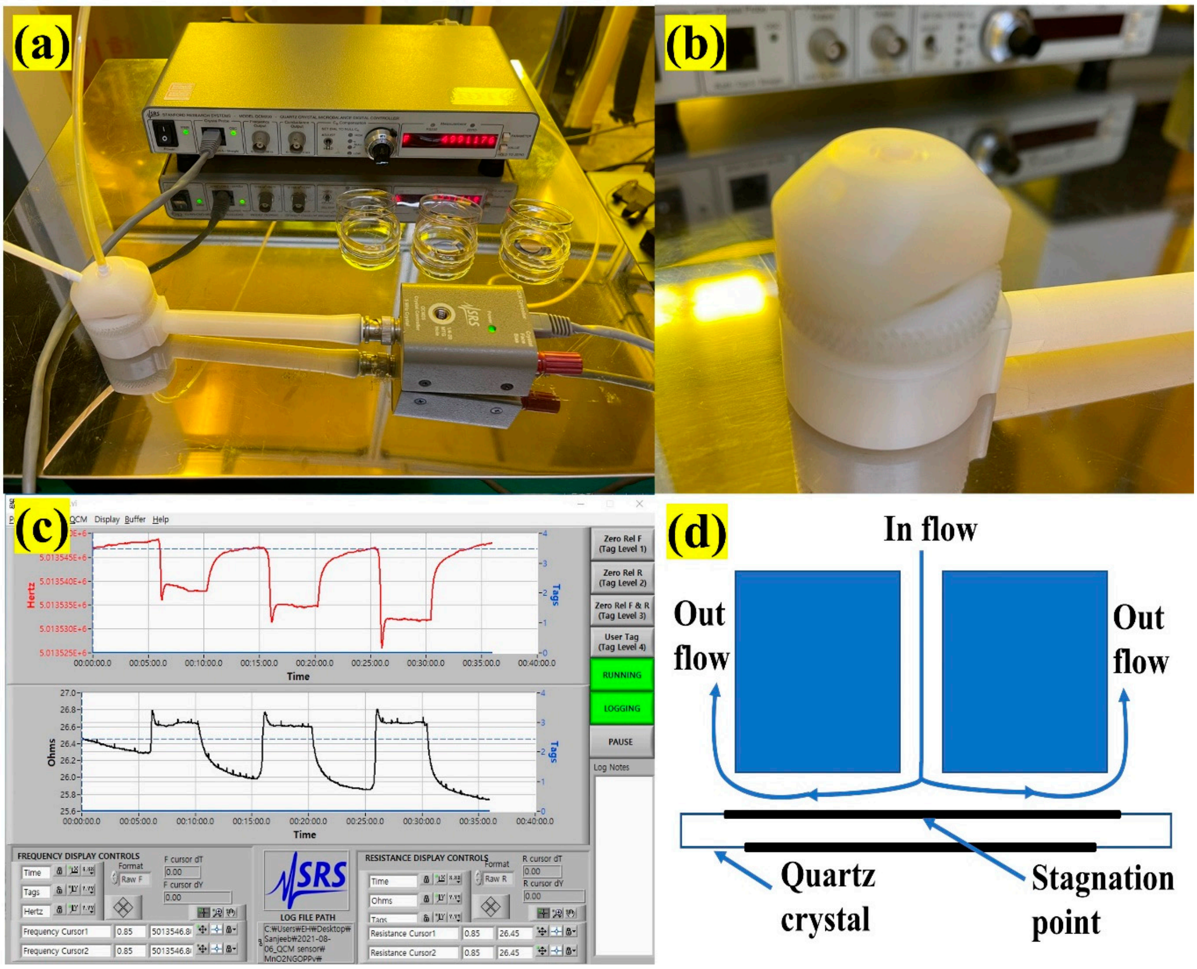

Figure S5. (a) QCM200 setup (digital controller, QCM crystals in Petri dish, crystal controller, and flow cell) (b) QCM flow cell (c) QCM signal monitoring program (d) Schematic diagram of gas flow pattern in the flow cell.

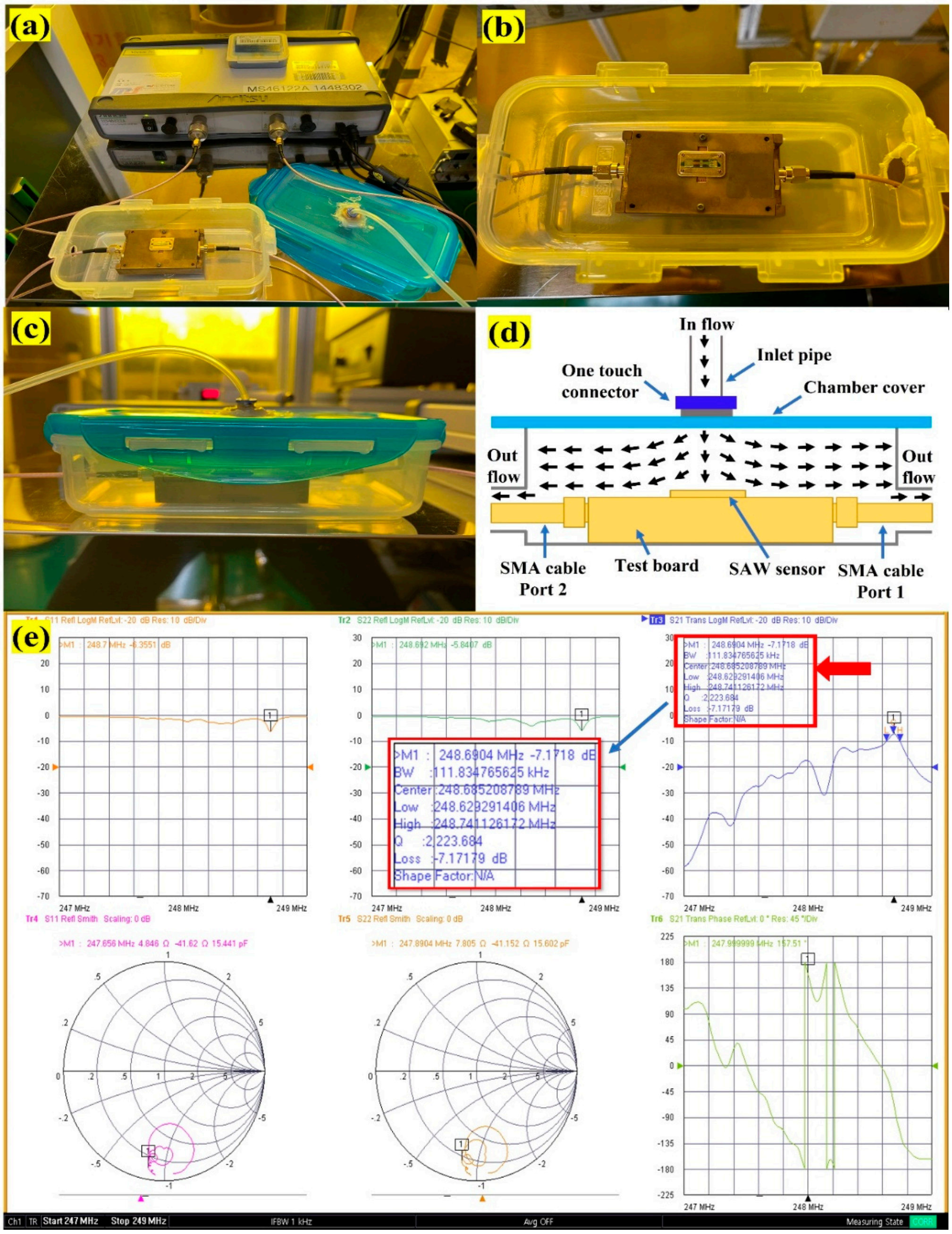

Figure S6. (a) SAW sensor setup for the experimentation (b) Top view of the SAW sensor mounted on the test board (c) Front view of detection chamber with top cover for gas monitoring (d) Schematic diagram of the gas flow diagram in the detection chamber (e) SAW sensor monitoring program to check signals from SAW ports.

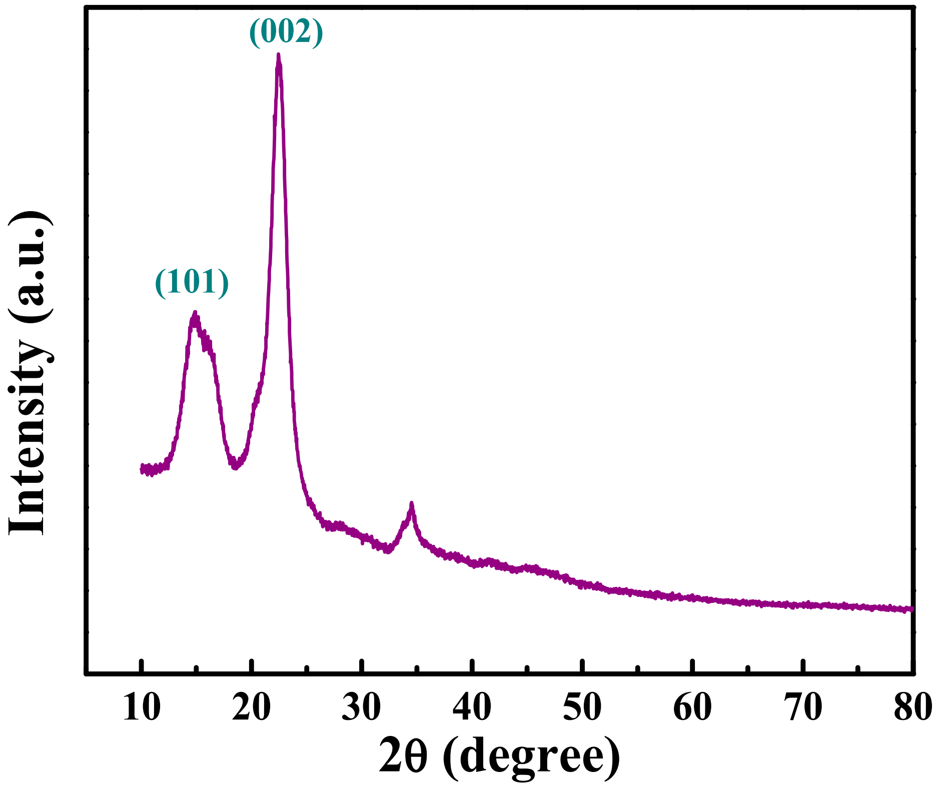

Figure S7. XRD analysis of cellulose.

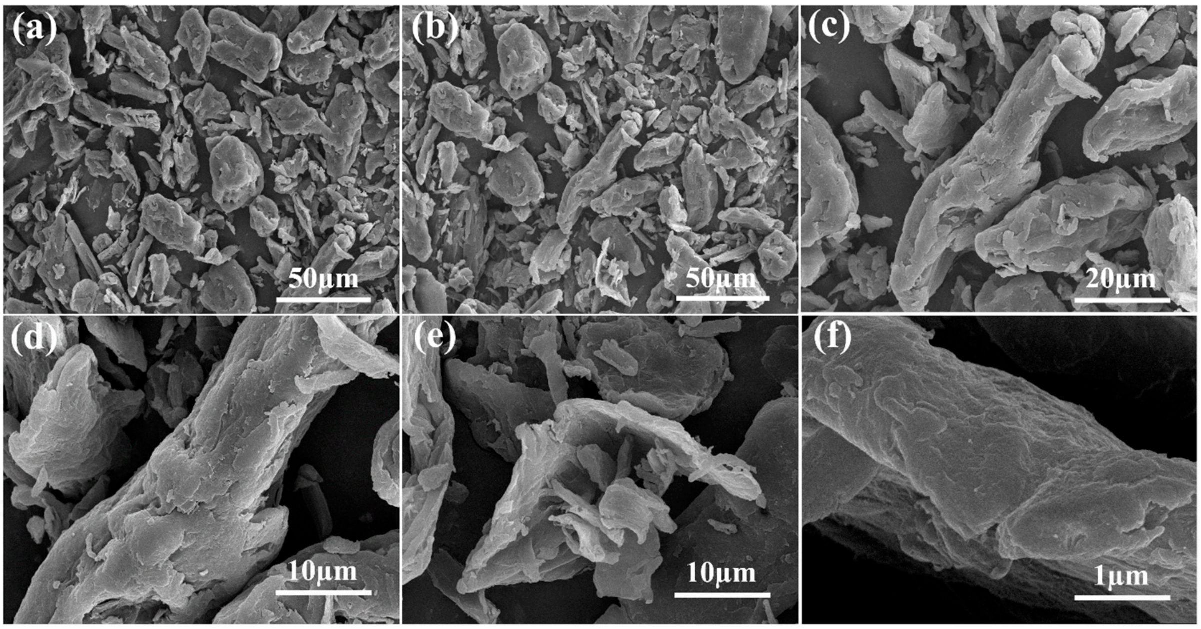

Figure S8. FE-SEM images of cellulose at the magnification of (a) 50 µm, (b) 50 µm, (c) 20 µm, (d) 10 µm, (e) 10 µm, and (f) 1 µm.

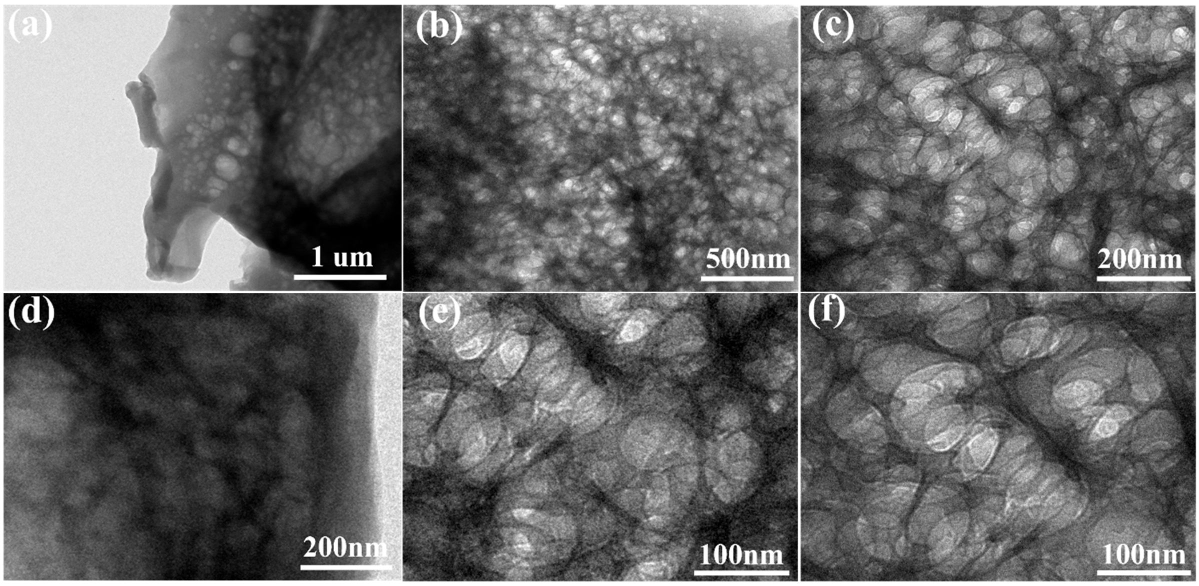

Figure S9. FE-TEM images of cellulose at the magnification of (a) 1 μm, (b) 500 nm, (c) 200 nm (d) 200 nm, (e) 100 nm, and (f) 100 nm.
